# Jittered echo-delay resolution in bottlenose dolphins (*Tursiops truncatus*)

**DOI:** 10.1007/s00359-018-1309-6

**Published:** 2018-12-26

**Authors:** James J. Finneran, Ryan Jones, Jason Mulsow, Dorian S. Houser, Patrick W. Moore

**Affiliations:** 1 U.S. Navy Marine Mammal Program, Space and Naval Warfare Systems Center Pacific, Code 71510, 53560 Hull St., San Diego, CA 92152 USA; 2 National Marine Mammal Foundation, 2240 Shelter Island Dr. #200, San Diego, CA 92106 USA

**Keywords:** Dolphin, Jitter, Echolocation, Biosonar, Target ranging

## Abstract

Psychophysical methods similar to those employed with bats were used to examine jittered echo-delay resolution in bottlenose dolphins (*Tursiops truncatus*). Two dolphins were trained to produce echolocation clicks and report a change from electronic echoes with a fixed delay of ~ 12.6 ms (~ 9.4 m simulated range) to echoes with delays that alternated (jittered) between successive emitted signals. Jitter delays varied from 0 to 50 µs. Jittered echo-delay thresholds were between 1 and 2 µs—the lowest achievable (non-zero) values with the hardware configuration. Error functions matched the click autocorrelation function near zero jitter delay, and were well within the envelope of the autocorrelation function; however, measured jitter delay thresholds were larger than predictions for a coherent or semicoherent receiver at comparable signal-to-noise ratios. When one of the two alternating jittered echoes was inverted in polarity, both dolphins reliably discriminated echoes at all jittered echo delays, including 0 µs (i.e., only jittering in polarity, not delay). Finally, both dolphins used unusual patterns of click emissions, where groups of echolocation clicks were interspersed with silent gaps. Further tests with sub-microsecond jitter values and various echo signal-to-noise ratios would be necessary for proper direct comparison with jitter detection values obtained for bats.

## Introduction

Both dolphins and bats emit high-frequency, directional sounds and perceive objects from echoes returned to their ears. Echoes from most objects have complex structures containing multiple “highlights” arising from combinations of specular reflections from the front surface, reflections from different parts of the object, internal reflections propagating along different paths, and contributions from circumferential waves traveling around the object (Au 1993). An important aspect of biosonar involves the perception and discrimination of the structure and timing relationships between target echo returns; i.e., the ability to separately perceive and locate closely spaced reflecting points or surfaces. The basis for this capability is resolution of the delays of closely spaced echoes (Simmons et al. 1990b). The range to an initial target highlight is coded by the elapsed time, or delay, between sound emission and echo return. However, subsequent highlights that fall within the integration time of the ear are represented by notches in the echo spectrum that have frequency spacing inversely proportional to echo highlight time separation. A neural process equivalent to frequency-to-time transform has been suggested to represent target range for these later highlights in echoes (Simmons et al. 1990b).

Experiments to study echo-delay resolution in bats have been conducted using a two-alternative, forced-choice paradigm where bats were conditioned to report which of two physical targets, arranged at different azimuthal angles, was closer (reviewed by Simmons and Grinnell 1988). The resulting range discrimination thresholds (75% correct) were generally within the range of 1–2 cm (~ 60–120 µs of echo delay) for the big brown bat (*Eptesicus fuscus*), lesser mouse-eared bat (*Myotis oxygnathus*), lesser bulldog bat (*Noctilio albiventris*), greater spear-nosed bat (*Phyllostomus hastatus*), common pipistrelle (*Pipistrellus pipistrellus*), and big naked-backed bat (*Pteronotus suapurensis*), and 3–4 cm (~ 180–240 µs of echo delay) for the greater horseshoe bat (*Rhinolophus ferrumequinum*) (see Simmons and Grinnell 1988). Studies were also conducted with similar procedures using phantom echoes rather than physical targets and yielded similar discrimination thresholds; e.g., Masters and Jacobs (1989) reported range discrimination thresholds of 0.7–1.7 cm (~ 40–100 µs echo delay) in *Eptesicus*.

Using methods similar to those used with bats, Murchison (1980) measured range discrimination thresholds in a bottlenose dolphin (*Tursiops truncatus*). In a two-alternative, forced-choice paradigm, the dolphin reported which of two identical foam spheres was closer. The targets were arranged at azimuthal angles of 20° and − 20° relative to the dolphin’s longitudinal axis and mean ranges of 1, 3, and 7 m. Range discrimination thresholds (75% correct) varied with absolute target range: 0.9, 1.5, and 3 cm for ranges of 1, 3, and 7 m, respectively (Murchison 1980). In terms of echo delay, thresholds were approximately 12, 20, and 40 µs for 1, 3, and 7 m, respectively.

A limitation of the range discrimination experiments described above is the potential error introduced by movement of the animal’s head as it scans the two targets (or phantom echo receivers) located at different azimuths (Simmons and Grinnell 1988). As a result, the “true” echo-delay resolution would be smaller than that estimated from the range discrimination experiment. For example, Simmons et al. (1990a) estimated that when head movements were taken into account, range discrimination thresholds for *Eptesicus* reduce from 1.3–1.4 cm to 0.5–0.7 cm (~ 30–40 µs of echo delay).

Experimental procedures to investigate fine-scale echo-delay resolution and avoid the interfering effects of head movement have been developed and tested in bats (Menne et al. 1989; Moss and Schnitzler 1989; Simmons 1979; Simmons et al. 1990a). The paradigm has been referred to as “jittered echo-delay acuity”. In these studies, big brown bats were trained to discriminate between electronic echoes with a fixed delay (i.e., simulating fixed range) and echoes with a delay that alternated (jittered) on successive presentations. The data consisted of the bats’ performance as a function of the amount of jitter in the echo delay (the time interval over which the echoes jittered). Simmons (1979) found that the bats’ performance as a function of jitter delay matched the half-wave-rectified cross-correlation (XCR) function between the emitted signal and received echo. These findings were replicated by Simmons et al. (1990a), who also reported a fine jitter delay acuity of 10 ns and attributed this result to temporal acuity rather than spectral acuity. These data are extraordinary because they suggest that bats can extract information from within the envelope of the XCR function; however, these results are controversial. Other investigators—using slightly different methodologies—obtained sub-microsecond resolution (testing was limited to hundreds of nanoseconds) but failed to observe reduced performance at jitter delays near the first positive peak in the XCR function of echoes (Menne et al. 1989; Moss and Schnitzler 1989; but see; Simmons et al. 1990a for possible explanation). Alternative potential explanations for the 10-ns jitter delay resolution have been proposed (and rebutted, see Simmons 1993; Simmons et al. 2003), including spectral artifacts caused by overlap between vocalizations, stimulus echoes, and extraneous sounds (Pollak 1993), and signal distortion from impedance mismatches in the delay-generating apparatus (Beedholm and Mohl 1998).

Experiments comparable to the bat-jittered-echo delay acuity experiments have not been conducted with dolphins or other echolocating odontocetes (toothed whales), and the extent to which dolphins utilize signal processing mechanisms similar to bats is unknown. This paper presents the results of a study examining echo-delay resolution in dolphins using a modified version of the jittered echo-delay acuity procedures previously used with bats (Menne et al. 1989; Moss and Schnitzler 1989; Simmons 1979; Simmons et al. 1990a). The primary goals were to determine if dolphins possess echo-delay resolution in the microsecond range, rather than the tens of microseconds previously determined with the range discrimination method (Murchison 1980), and how performance curves (i.e., decrements in jitter detection capabilities at specific jitter delay values) compare with click and echo autocorrelation (ACR) and XCR functions.

## Methods

### Subjects and test environment

Subjects consisted of two bottlenose dolphins: SAY (female 37 years) and APR (female 32 years). Upper-cutoff frequencies for their hearing, defined as the frequency at which psychophysical thresholds reached a sound pressure level (SPL) of 100 dB re 1 µPa, were ~ 110 kHz for APR and ~ 140 kHz for SAY, indicating full hearing bandwidth for SAY and limited high-frequency loss for APR.

**Fig. 1:**
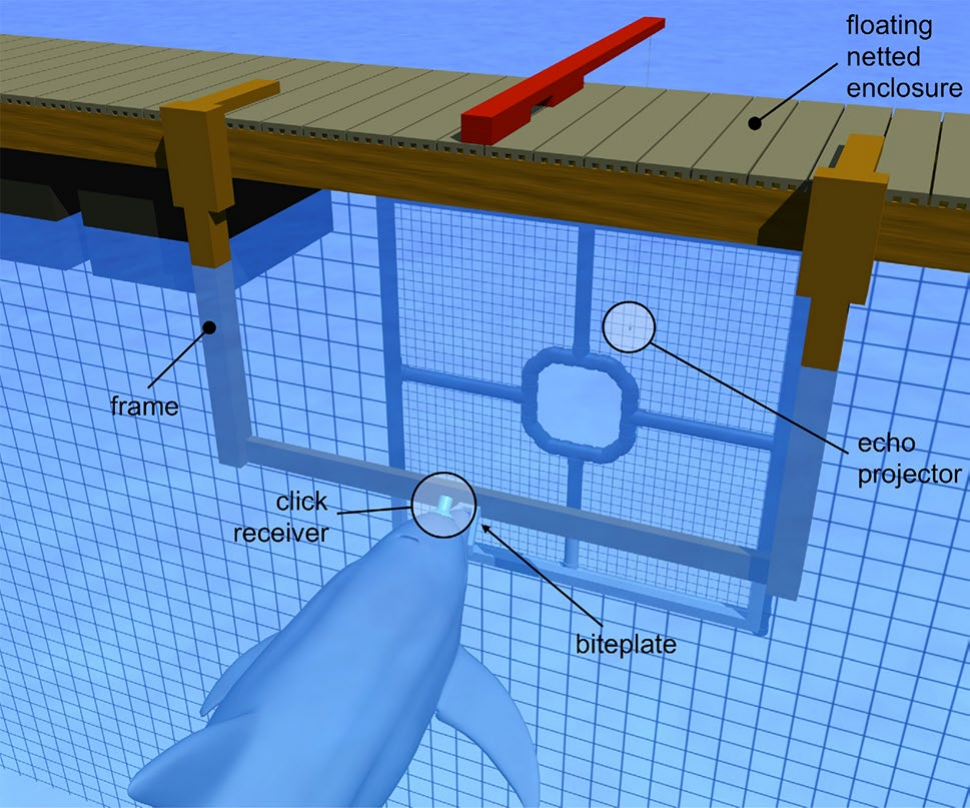
Experiments were conducted with a dolphin positioned on a “biteplate” apparatus suspended under water in San Diego Bay. Emitted dolphin biosonar clicks were recorded using a hydrophone (click receiver) embedded in a suction cup placed on the melon. The dolphin’s task was to discriminate between echoes with fixed delay (non-jittering) and those with delay alternated (jittered) from one click emission to the next

All tests were conducted within a 9 m × 9 m floating, netted enclosure at the US Navy Marine Mammal Program facility in San Diego Bay, California. During each trial, the dolphin positioned itself on an underwater “biteplate” supported at a depth of 80 cm by vertical posts spaced 1.8 m apart. The biteplate was oriented so the dolphin faced San Diego Bay through an enclosure gate opening containing a netted frame with a central observation aperture (Fig. 1). Beyond the gate opening, at a distance of 1.3 m, a piezoelectric transducer (TC4013, Reson Inc., Slangerup, Denmark) was positioned for use as the echo projector. The nearest land masses within ± 20° of the dolphin’s main biosonar transmission axis while on the biteplate were ~ 500-m distant. Mean water depth was ~ 4–5 m. Background ambient noise at the test site was dominated by snapping shrimp and other dolphins, with occasional contributions from passing vessels and aircraft.

### Task description

**Fig. 2:**
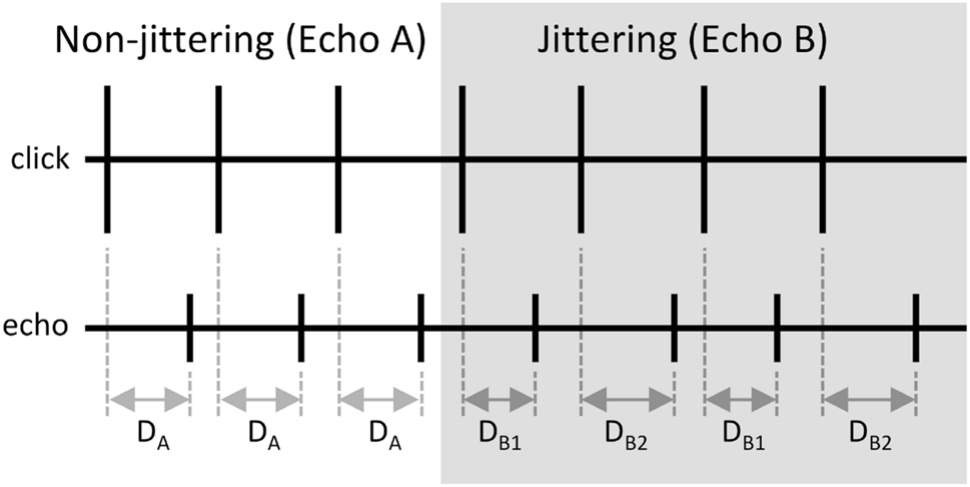
Relationship between the timing of emitted clicks (upper panel) and non-jittering and jittering echoes (lower panel). The lines represent instantaneous sound pressure. The horizontal axis represents time. Non-jittering echoes (Echo A) were generated at a fixed time delay (*D*_A_) after click emission. For jittering echoes (Echo B), the time delay between click emission and echo generation alternated (jittered) between *D*_B1_ on one click emission to *D*_B2_ on the next. For Exps. 1 and 3, *D*_A_ = (*D*_B1_ + *D*_B2_)/2. For Exp. 2, *D*_A_ = *D*_B1_. Jitter delay Δ*T* = *D*_B2_ − *D*_B1_

The biosonar task required the dolphin to produce echolocation clicks, listen to returning echoes created by convolving the received click with an impulse function, and produce a conditioned acoustic response (a whistle) when the echoes changed from non-jittering (Echo A) to jittering (Echo B). For non-jittering echoes, echo delay and echo polarity were fixed. For jittering echoes, echo delay and echo polarity could vary on alternate echoes (i.e., different values for even or odd numbered echoes within a trial, see Fig. 2). Three experiments were conducted: Experiments 1 and 2 featured jittered echo delay only and Exp. 3 featured jittered echo delay and echo polarity (Table 1). Non-jittering echo delay was fixed at 12.56 ms (~ 9.4 m simulated target range) for all experiments.

**Table 1:** Echo delay and polarity characteristics for the three experiments

	Echo A	Echo B		
		Exp. 1	Exp. 2	Exp. 3
Delay	*T*	*T* − Δ*T*/2 *T* + Δ*T*/2	*T* *T* + Δ*T*	*T* − Δ*T*/2 *T* + Δ*T*/2
Polarity	+ 1	+ 1 + 1	+ 1 + 1	+ 1 − 1

Jitter delay is represented by Δ*T*. Experiments 1 and 2 featured jittered delay only, but differed in the relationship between the jittering and non-jittering echo delays: Exp. 1 featured symmetric jitter, where the echo delay for the non-jittering echo equaled the mean of the echo delay for the jittering echoes. Exp. 2 featured asymmetric jitter, where the echo delay for the non-jittering echo equaled the shorter delay value of the two jittering delays. In Exp. 3, both echo delay and echo polarity (i.e., 180° phase shift) were jittered. Echo delay was jittered symmetrically in Exp. 3

Experimental sessions typically consisted of 60 trials and lasted ~ 30 min. Within each session, 80% of the trials were designated as echo change trials, where the echoes changed from non-jittering to jittering after a random interval of 5–10 s, followed by a 1-s response window. On the remainder of the trials (control trials), non-jittering echoes were presented for the entire 6–11-s trial duration. If the dolphin responded during the 1-s response interval after an echo change (a hit), or withheld the response for an entire control trial (a correct rejection), it was rewarded with one fish. The dolphin was recalled to the surface with no fish reward for responding during a control trial (a false alarm) or for failing to respond during a response interval following an echo change (a miss). If the dolphin responded before the echoes changed during an echo change trial, it was immediately recalled to the surface with no fish reward, and the trial was re-classified as a control trial and scored as a false alarm. If the dolphin did not echolocate during a trial, stopped echolocating before the echoes changed, left the biteplate, or was visually observed to be echolocating on another object, it was recalled and the trial data were discarded.

During data collection sessions, jitter delay varied from 50 µs down to 2, 1, or 0 µs for Exps. 1, 2, and 3, respectively (initial training utilized much larger jitter delay values). Sessions were divided into 10-trial blocks with constant jitter delay within each block. Each session typically featured six jitter delay values, which were tested in descending order. Within each experiment, at least 40 echo-change trials were conducted for each value of jitter delay; this required 21–23 sessions for Exp. 1 and 9 to 10 sessions for Exps. 2 and 3 (Exps. 2 and 3 featured fewer values of jitter delay).

### Echo generation

**Fig. 3:**
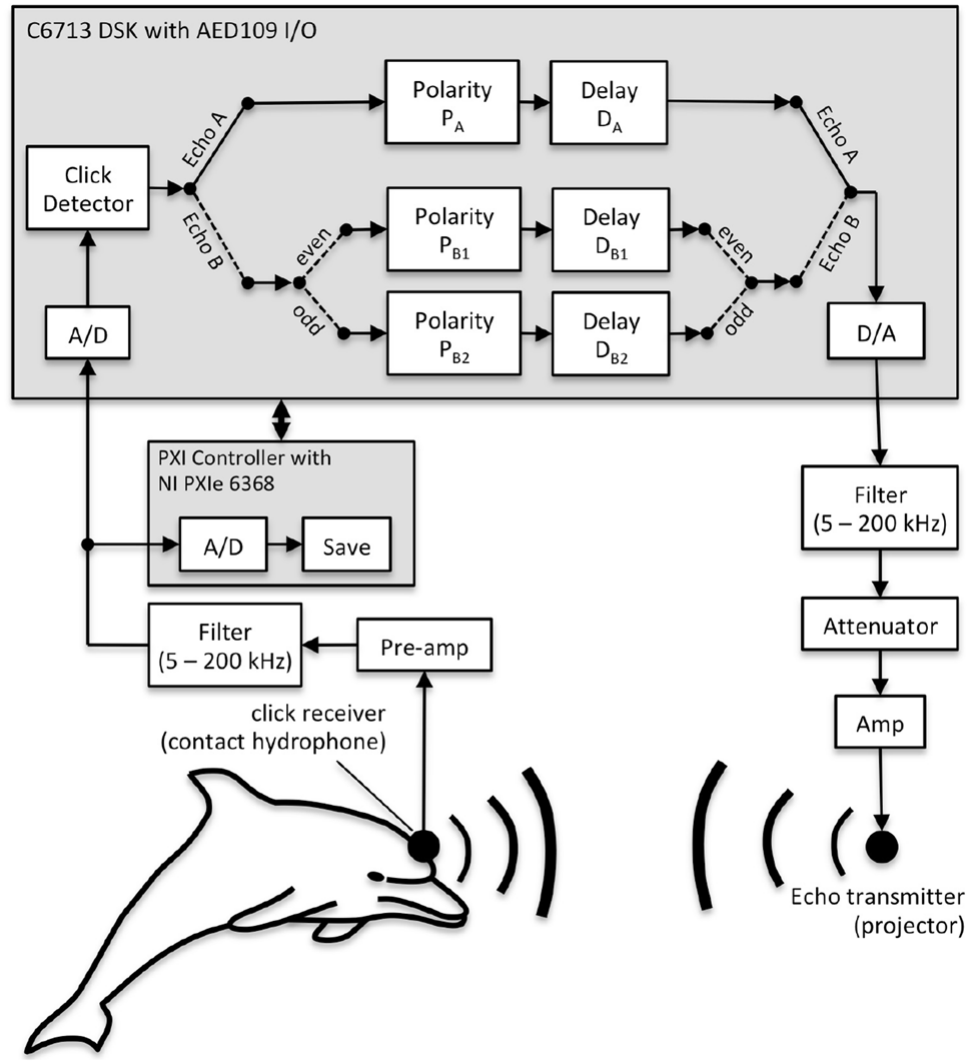
Echoes were synthesized using a phantom echo generator based on a C6713 digital signal processor. Received clicks that exceeded an amplitude threshold were digitized, delayed in time, scaled in amplitude, and broadcast to the dolphin via an underwater sound projector (echo projector). Echoes changed from non-jittering (Echo A) to jittering (Echo B) on 80% of the trials, after a random interval of 5–10 s

Biosonar echoes were created using a phantom echo generator (PEG, Fig. 3) based on a TMS320C6713 floating point digital signal processor (Texas Instruments, Dallas, TX) with an analog input/output (I/O) daughtercard (AED109, Signalware Corp., Colorado Springs, CO). The system operated in an “open-loop” fashion, where click signals that exceeded a threshold triggered the creation of echo waveforms that were then held in digital memory before transmission to the dolphin. This operating mode is in contrast to a digital delay line that would simply delay all received signals and transmit back to the dolphin. Clicks emitted by the dolphin were recorded using a hydrophone (TC4013, Reson Inc., Slangerup, Denmark) embedded in a silicon suction cup and attached to the dolphin’s melon along the main biosonar transmission axis. This arrangement minimized potential echo timing errors associated with head movements. The contact hydrophone signal (Fig. 3) was amplified and filtered (5–200 kHz bandwidth: VP-1000, Reson Inc., Slangerup, Denmark and 3C module, Krohn-Hite Corporation, Brockton, MA), then digitized by the AED109 with a 1-MHz sampling rate and 12-bit resolution. The digitized hydrophone signal was passed to a threshold-crossing click detector. If a click was detected, the click waveform was scaled in amplitude, delayed by the appropriate time, then converted to analog (AED109). The outgoing analog waveform was filtered (5–200 kHz, 3C module, Krohn-Hite Corporation, Brockton, MA), attenuated if necessary (PA5, Tucker-Davis Technologies, Alachua, FL), amplified (M7600, Krohn-Hite Corp.), and used to drive the echo transmitter. The echo level relative to the emitted click—not the absolute echo level—was constant; i.e., echo levels dynamically changed in response to changes in emitted click level. Energy flux density levels of echo A and B were approximately − 72 dB relative to the received click at the contact hydrophone (about 20 dB above echo-detection threshold). The dolphin clicks and electronic echo waveforms were digitized at 2 MHz and 16-bit resolution by a PXIe-6368 multifunction data acquisition device (National Instruments, Austin, TX) and stored for later analysis.

Ambient noise was monitored using a hydrophone (TC4032, Reson Inc.) located ~ 50 cm above and to the side of the biteplate. The signal from this “off-axis” hydrophone was high-pass filtered at 100 Hz before being digitized at 2 MHz and 16-bit resolution by the same PXIe-6368 used for click recording.

Changes in echo delay (i.e., jitter in echo delay) were accomplished by changing the position of the echo waveform in the 1-MHz digital-to-analog (D/A) converter output buffer on an echo-by-echo basis. This approach had the advantage of preventing delay-dependent changes in echo amplitude and spectral content, but echo-delay resolution was limited to a single sample interval (1 µs). Experiments 1 and 3 featured echo delays that symmetrically jittered about the non-jittering echo delay (Table 1); therefore, the PEG D/A sampling rate restricted jitter delays to integral multiples of 2 µs. For Exp. 2, the shorter of the jittering delay values matched the non-jittering delay; therefore, the minimum jittered delay resolution was 1 µs.

Operation of the PEG was verified before each session by replacing the dolphin click signal input to the PEG analog-to-digital (A/D) converter with an electronic signal resembling a dolphin click and inspecting the resulting electronic echo waveform. A high-speed digital oscilloscope was used to ensure the actual jitter delay values matched the desired values. Calibration measurements using the oscilloscope revealed potential errors (i.e., unavailable, random jitter) in echo delay of less than ± 15 ns for the electronic echoes (i.e., before transmission into the water). Potential movement of the dolphin relative to the echo projector (not more than approximately 3 cm on a trial) could have caused larger changes in echo delay. However, as movement would have occurred on a relatively slow time scale compared to the changes arising from jittering echo delay from one echo to the next, the effect was considered to be negligible.

### Analysis

Each dolphin’s performance in the echolocation task was quantified using the hit rate:

1 $$H={N_H}/{N_{{\text{EC}}}},$$

false alarm rate

2 $$F={N_{{\text{FA}}}}/{N_{{\text{NC}}}},$$

and the error rate (i.e., the number of incorrect responses divided by total number of trials)

3 $$E=(1 - H)\frac{{{N_{{\text{EC}}}}}}{N}+F\frac{{{N_{{\text{NC}}}}}}{N},$$

where *H* is the hit rate, *F* is the false alarm rate, *E* is the error rate, *N*_*H*_ is the number of hits, *N*_FA_ is the number of false alarms, *N*_EC_ is the number of echo-change trials, *N*_NC_ is the number of control trials, and *N* = *N*_EC_ + *N*_NC_ is the total number of trials. The error rate is used here to facilitate comparison with bat-jittered echo delay results presented as error rates from the two alternative forced choice paradigm (e.g., Simmons 1979).

Ambient noise during experimental sessions was quantified by computing the pressure spectral density from the off-axis hydrophone signal over 4096-sample (~ 2 ms) time intervals just before the generation of each echo. Biosonar click emissions were quantified by extracting emitted clicks from the digitized contact hydrophone signal, then computing the 10, 50, and 90th amplitude percentiles at each time value. Echo calibration was done with representative echoes, obtained by replacing the contact hydrophone input to the PEG with a waveform representing the median of the recorded clicks, then measuring the acoustic echo waveform projected back to the listening position (midpoint between the dolphin’s lower jaws) without the dolphin present. This allowed the use of coherent averaging to extract echoes from the background ambient noise. Echo waveforms were temporally aligned during averaging using the time delay of the peak of the cross-correlation function between each echo and the mean of the preceding (previously aligned) echoes (Woody 1967). Representative examples of farfield dolphin clicks were also obtained from recordings made during preliminary training/testing with a hydrophone located at a distance of 1 m on the biosonar main transmit axis. For representative clicks and echoes, the peak–peak sound pressure level (SPL), energy flux density level, energy spectrum, and the ACR function were calculated. For comparison to the click/echo ACR functions, the XCR function between the farfield click and echo was also computed. Envelopes for ACR and XCR functions were derived using the magnitude of the analytic function whose real part is the function itself and imaginary part is the Hilbert transform of the function (Au 1993).

For optimal receivers, maximum accuracy in echo delay estimation (minimum standard deviation of the delay estimate, *σ*) can be expressed as *σ* = (2*πBd*)^−1^, where *B* is the echo bandwidth, *d* = $\sqrt {2E/{N_0}}$ is the echo amplitude signal-to-noise ratio (SNR), *E* is the echo energy flux density, and *N*_0_ is the noise pressure spectral density (Menne and Hackbarth 1986; Schnitzler et al. 1985; Simmons et al. 2004). The definition of bandwidth varies depending on whether the receiver is coherent or semicoherent. For a coherent receiver, echo delay is estimated using the maximum peak in the fine structure of the XCR function and bandwidth is given by the rms bandwidth, *B*_rms_:

4 $${B_{{\text{rms}}}}={\left( {\frac{{\int_{{ - \infty }}^{\infty } {{f^2}{{\left| {S(f)} \right|}^2}{\text{d}}f} }}{{\int_{{ - \infty }}^{\infty } {{{\left| {S(f)} \right|}^2}{\text{d}}f} }}} \right)^{1/2}},$$

where *f* is frequency and *S*(*f*) is the Fourier transform of the signal waveform (Au 1993; Menne and Hackbarth 1986; Simmons et al. 2004). For a semicoherent receiver, echo delay is estimated using the peak of the envelope of the XCR function and bandwidth is defined by the centralized rms bandwidth (*B*_crms_):

5 $${B_{{\text{crms}}}}={\left( {\frac{{\int_{{ - \infty }}^{\infty } {{{\left( {f - {f_{\text{c}}}} \right)}^2}{{\left| {S(f)} \right|}^2}{\text{d}}f} }}{{\int_{{ - \infty }}^{\infty } {{{\left| {S(f)} \right|}^2}{\text{d}}f} }}} \right)^{1/2}}=\sqrt {B_{{{\text{rms}}}}^{2} - f_{{\text{c}}}^{2}} ,$$

where the center (centroid) frequency (*f*_c_) is defined as:

6 $${f_{\text{c}}}=\frac{{\int_{{ - \infty }}^{\infty } {f{{\left| {S(f)} \right|}^2}{\text{d}}f} }}{{\int_{{ - \infty }}^{\infty } {{{\left| {S(f)} \right|}^2}{\text{d}}f} }},$$

(Au 1993; Menne and Hackbarth 1986; Simmons et al. 2004).

## Results

### Performance

**Fig. 4:**
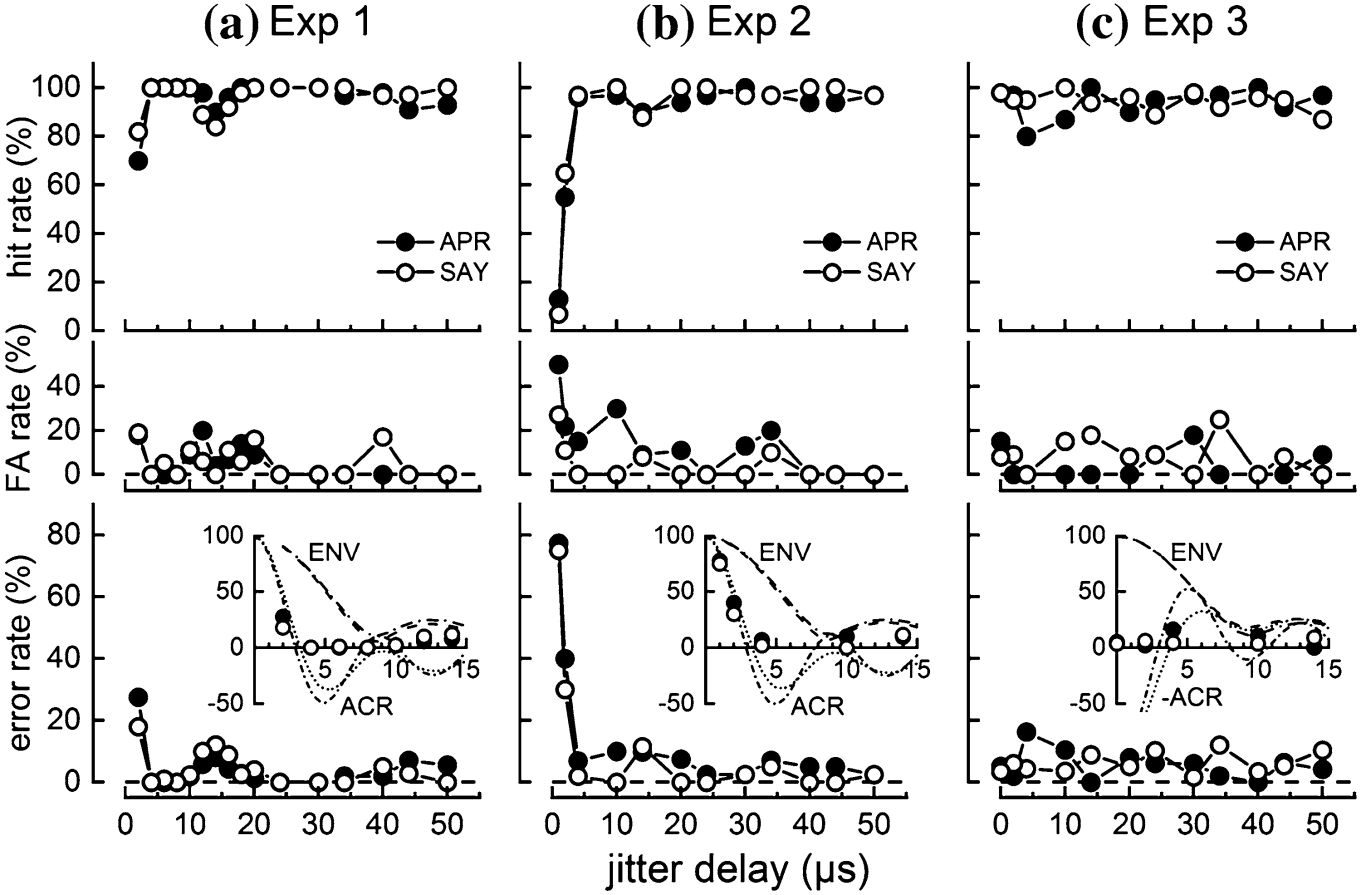
Performance of the dolphins APR and SAY during jittered echo discrimination tasks with **a** Experiment 1—symmetric echo delay jitter, **b** Experiment 2—asymmetric echo delay jitter, and **c** Experiment 3—symmetric echo delay and echo polarity jitter. Dolphin performance is shown in terms of the hit rate (upper panels), false alarm (FA) rate (middle panels), and error rate (lower panels). The inset graph in the lower panels compares the error rate from 0 to 15 µs to the autocorrelation (ACR) function and ACR envelope (ENV) of the click recorded by the contact hydrophone. For Exp. 3, the ACR functions are negated to reflect the cross-correlation between normal and 180° phase-shifted clicks. In the inset graphs, the ENV and ACR functions use dotted lines for APR and dot-dash lines for SAY. For Exps. 1 and 2, hit rate decreased and error rate increased for jitter delays ≤ 2 µs and between 14 and 16 µs. For Exp. 3, error rates were < 20% at all values of jitter delay, including zero

Figure 4 shows the dolphins’ performance during Exps. 1–3. Both dolphins discriminated between echoes with jittering and non-jittering echo delay with low error rates (≤ 12%) when jitter delay was ≥ 4 µs (Fig. 4a, b). Error rates rose to ~ 20–40% for a jitter delay of 2 µs and to ~ 80% for a delay of 1 µs (Exp. 2). The error peaks centered at 0 µs had widths of ~ 2–4 µs, well below the width of the envelope of the click ACR function but closely matching the ACR function itself (Fig. 4 insets). Data for both dolphins also exhibited a consistent drop in hit rate (and thus higher error rate) for jitter delays near 14 µs compared to that at 10 and 20 µs, though the overall hit rates did not drop below 84%. When both echo delay and echo polarity were jittered (Fig. 4c), error rates for both dolphins were low (< 20%), even when jitter delay was zero. In other words, both dolphins were able to discriminate echoes that differed only in polarity.

### Acoustic signals

**Fig. 5:**
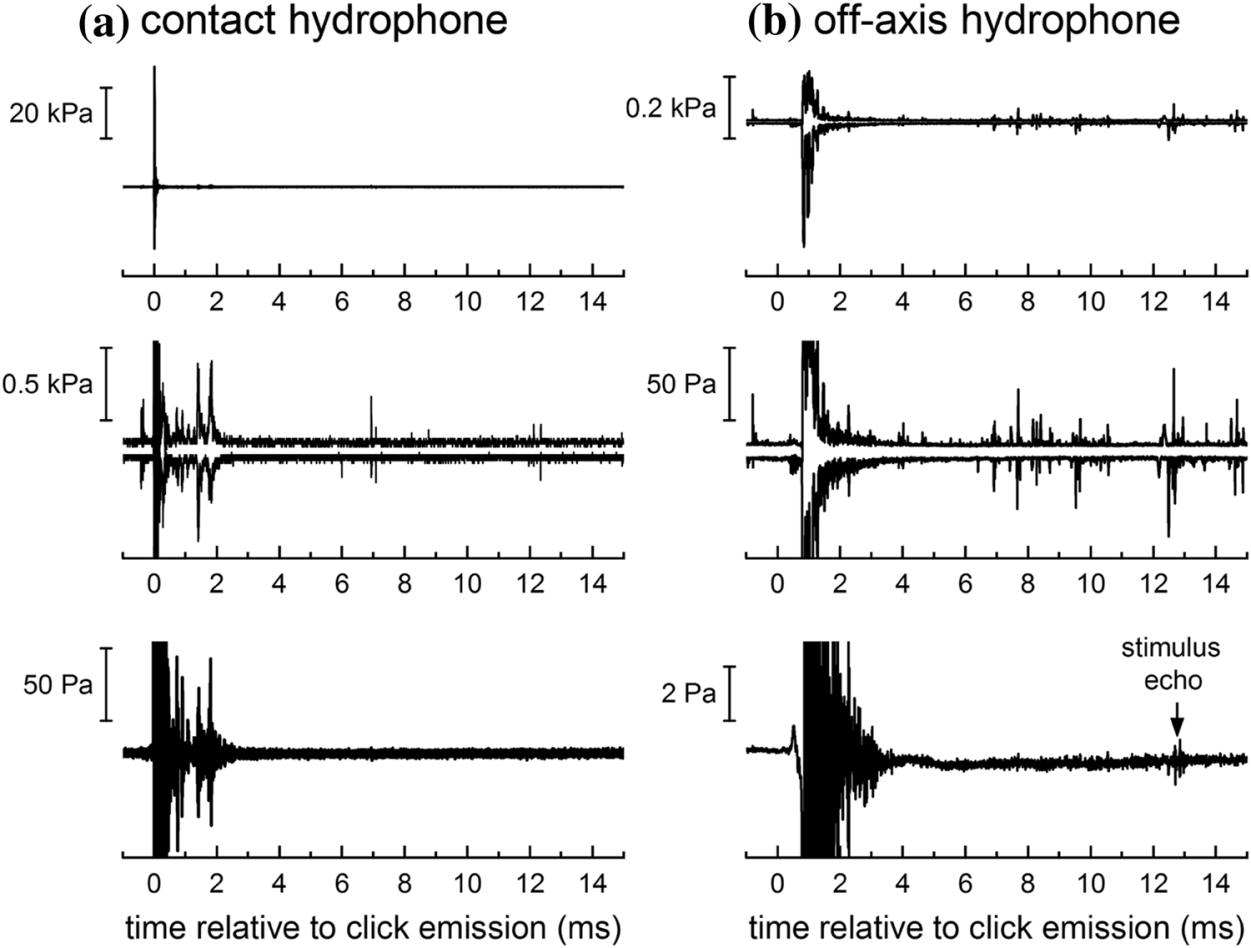
Instantaneous sound pressure recorded on the **a** contact hydrophone and **b** off-axis hydrophone over 16-ms time periods temporally aligned with the emitted clicks from a single, representative trial. The top and middle panels show the same data—the maximum and minimum sound pressure at each time sample—but with different *y*-axis scale factors. The lower panels show the mean sound pressure at each time sample. Impulsive signals from snapping shrimp or other dolphins are visible in the minimum/maximum data; however, the mean sound pressures reveal no visible reflections/reverberation from the apparatus and netted enclosures beyond ~ 4 ms relative to click emission

Critical evaluations of previous jitter experiments in bats have examined the potential for cues originating from temporal overlap between the bats’ emissions and stimulus echoes and/or echoes from objects in the environment (Pollak 1993; Simmons 1993). To assess the potential for temporal overlap of clicks and echoes in the present experiments, 16-ms time segments temporally aligned with each emitted click were extracted from the contact and off-axis hydrophone data from individual trials. Figure 5 shows the maximum and minimum sound pressures (top row, with middle row showing expanded *y*-axis for detail) and the mean sound pressure (bottom row) from a representative trial. Although the minimum/maximum data show occasional impulsive signals from snapping shrimp or other dolphins, the mean sound pressure reveals no visible reflections/reverberation from the apparatus and netted enclosures beyond ~ 4 ms relative to click emission. Given the short duration of dolphin clicks, large mean stimulus echo delay (12.56 ms), and the absence of any reflecting surfaces at sufficient distance to create reflections with comparable echo delays, the potential for consistent direct overlap of stimulus echoes with the emitted click or reflections from static objects in the environment was negligible.

**Fig. 6:**
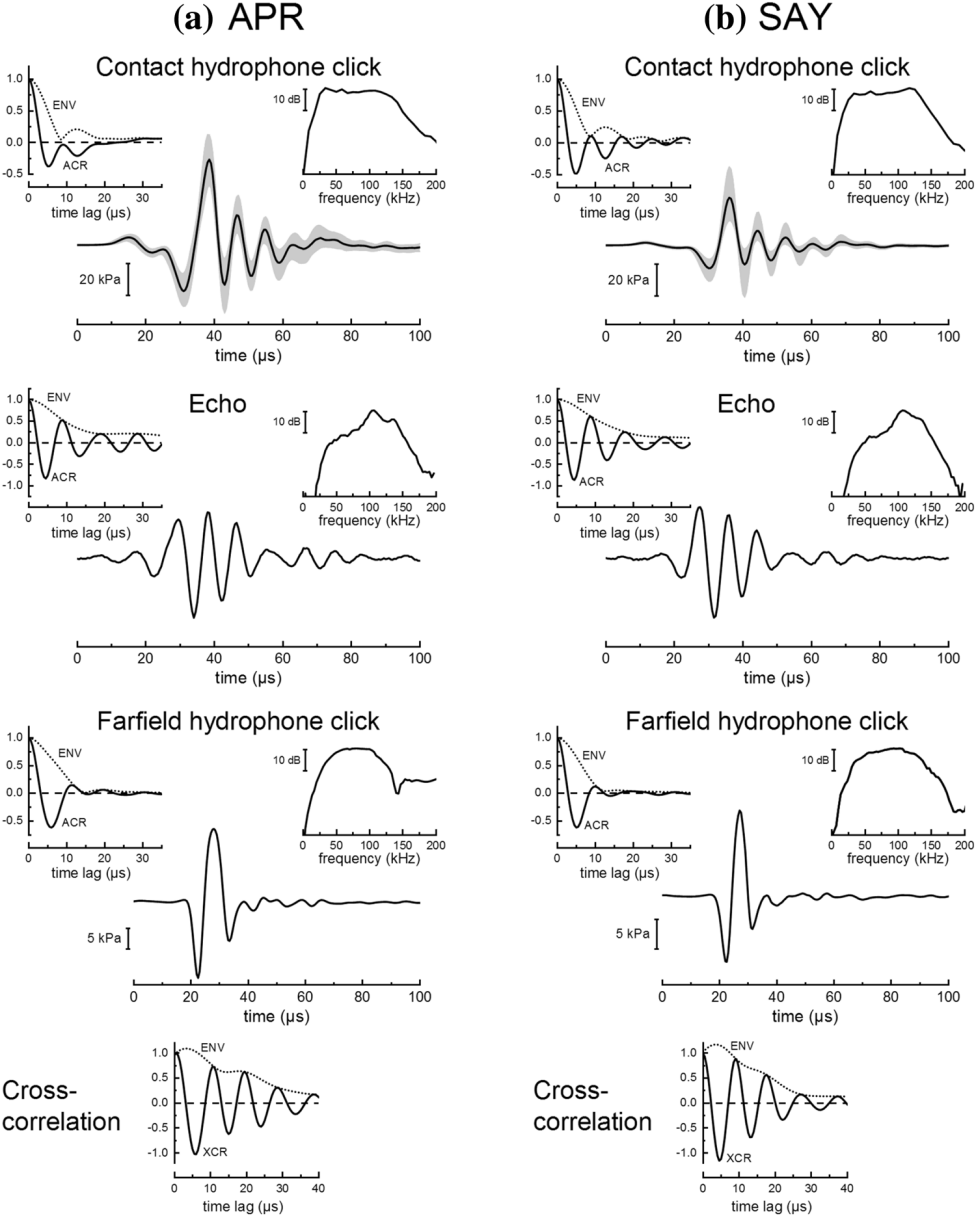
Representative waveforms, spectra, and autocorrelation (ACR) functions for clicks recorded by the contact hydrophone, acoustic echoes received by the dolphins, and clicks recorded in the acoustic farfield, for the dolphins **a** APR and **b** SAY. For the contact hydrophone clicks, the thick line shows the median sound pressure and the shaded region indicates the 10th and 90th percentiles. The spectra and ACR functions are based on the median click waveform. For calibration purposes, echo waveforms were obtained by generating phantom echoes in response to the median contact hydrophone click, then measuring the acoustic echo waveform projected back to the listening position (midpoint between the dolphin’s lower jaws, without the dolphin present). Representative farfield clicks were measured during preliminary testing. The bottom panel shows the cross-correlation (XCR) between the farfield click and echo. Envelopes for ACR and XCR functions were derived using the magnitude of the analytic function whose real and imaginary parts are composed of the function itself and the Hilbert transform of the function, respectively

Clicks recorded at the contact hydrophone (Fig. 6) resembled exponentially damped sinusoids with frequency content up to ~ 150 kHz and were similar to those typically measured in the farfield along the main biosonar transmit axis. Emitted clicks for SAY tended to have higher amplitudes and center frequencies compared to those emitted by APR, but both animals utilized similar click bandwidths. Across the three experiments, mean p–p SPLs and energy flux density levels at the contact hydrophone were 216–219 dB re 1 µPa and 160–162 dB re 1 µPa^2^s, respectively, for APR and 213–215 dB re 1 µPa and 156–158 dB re 1 µPa^2^s, respectively, for SAY. Mean values across the three experiments for the click *f*_c_, *B*_rms_, and *B*_crms_ ranged from 76 to 83 kHz, 83 to 92 kHz, and 33 to 35 kHz, respectively, for APR, and 86–104 kHz, 93–110 kHz, and 32–34 kHz, respectively, for SAY. The frequency response of the echo projector resulted in echo waveforms with less low-frequency energy and longer durations, with a slower amplitude decay rate compared to the click. Estimated values for echo *f*_c_, *B*_rms_, and *B*_crms_ ranged from 106 to 117 kHz, 110 to 121 kHz, and 22 to 30 kHz, respectively, for APR and SAY.

**Fig. 7:**
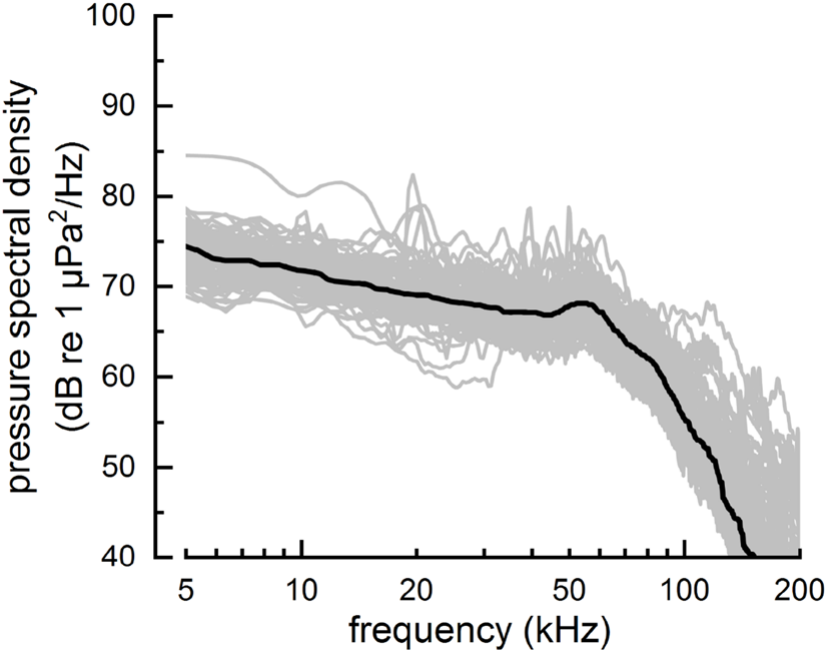
Ambient noise levels during experimental sessions were quantified by computing the pressure spectral density from the off-axis hydrophone signal over 4096-sample (~ 2 ms) time intervals, just before the generation of each echo. Each of the lighter traces shows the mean pressure spectral density for a single session. The thick line shows the smoothed median value

Over all experimental trials, the ambient noise pressure spectral density level (Fig. 7) averaged over a frequency range equal to the mean value for *f*_c_ ± *B*_crms_/2 was 57 dB re 1 µPa^2^/Hz (SD = 4.0 dB). When averaged over the entire nominal biosonar frequency range of 20–120 kHz, mean noise spectral density was 61 dB re 1 µPa^2^/Hz (SD = 4.2 dB). For typical click levels employed by APR and SAY, assuming an ambient noise pressure spectral density of 60 dB re 1 µPa^2^/Hz, mean values for echo SNR in dB (i.e., 20log_10_*d*) were ~ 25–35 dB. Using the estimated values for echo bandwidth and SNR = 25–35 dB, maximum delay accuracy would be ~ 25–80 ns for a coherent, optimal receiver and ~ 100–300 ns for a semicoherent receiver. To account for the change in noise spectral density with frequency when calculating SNR, echo energy flux spectral density values can be “weighted” by multiplying the echo energy flux spectral density by the reciprocal of the noise power spectral density. To prevent low values for noise spectral density resulting in large values for weighted energy flux densities at frequencies beyond the dolphins’ hearing ability, echo energy is also weighted by the dolphin’s auditory sensitivity curve (the inverse of the audiogram). Using this spectral weighting approach, SNR values increase to 43–55 dB and maximum delay accuracy reduces to ~ 2–10 ns for a coherent, optimal receiver and ~ 30–100 ns for a semicoherent receiver. In all cases, the maximum delay accuracy estimated for optimal receivers is well below the echo-delay thresholds measured in the present study (and the capabilities of the experimental hardware).

### Click emission patterns

**Fig. 8:**
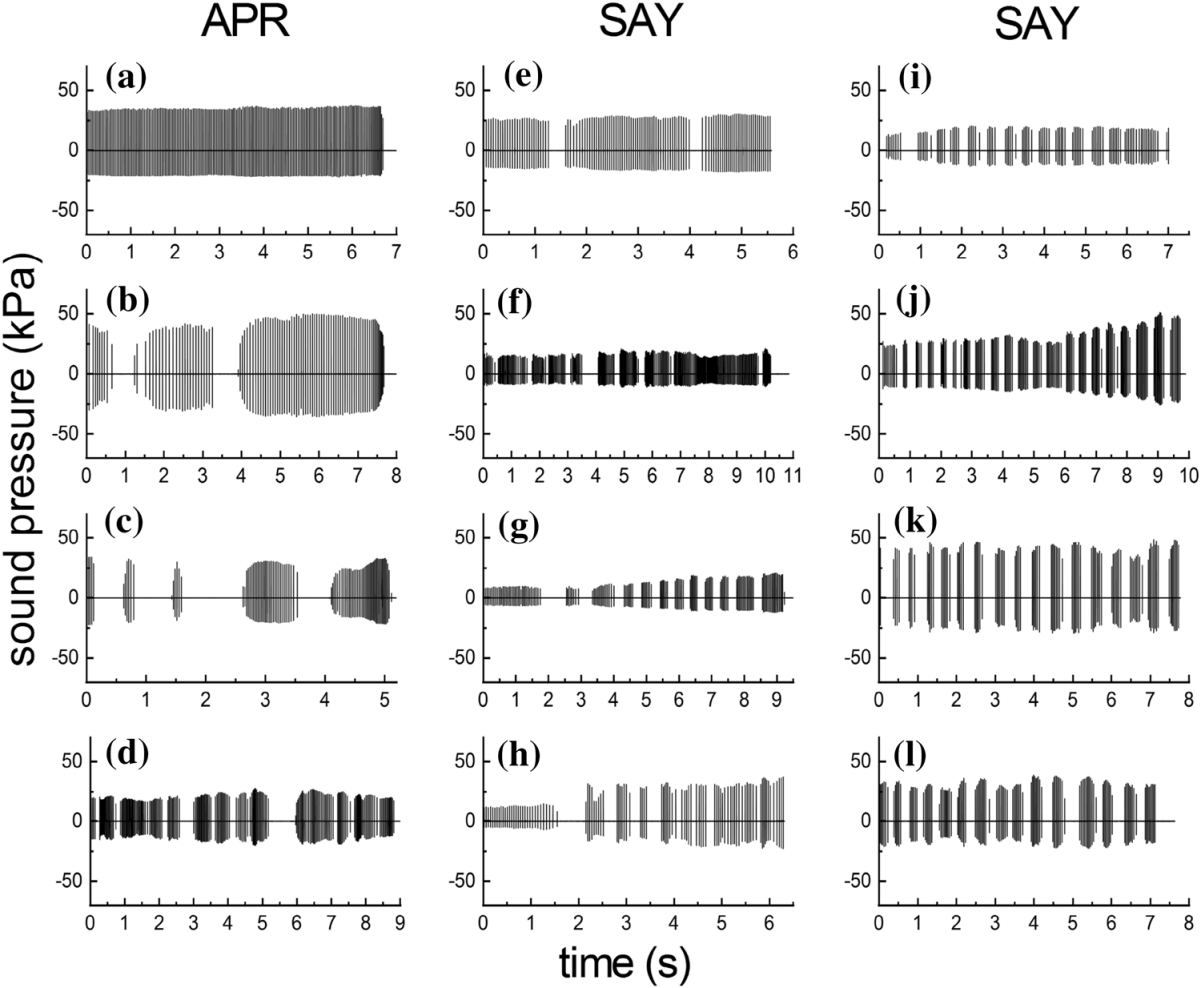
Representative click emission patterns for the dolphins APR (left column) and SAY (right columns). APR typically produced continuous sequences of clicks with no temporal gaps as in **a**, but occasionally utilized intermittent click groups (ICGs) with relatively large temporal gaps separating groups of clicks (**b**–**d**). SAY almost always utilized ICGs, examples of which are illustrated in **e**–**l**

**Fig. 9:**
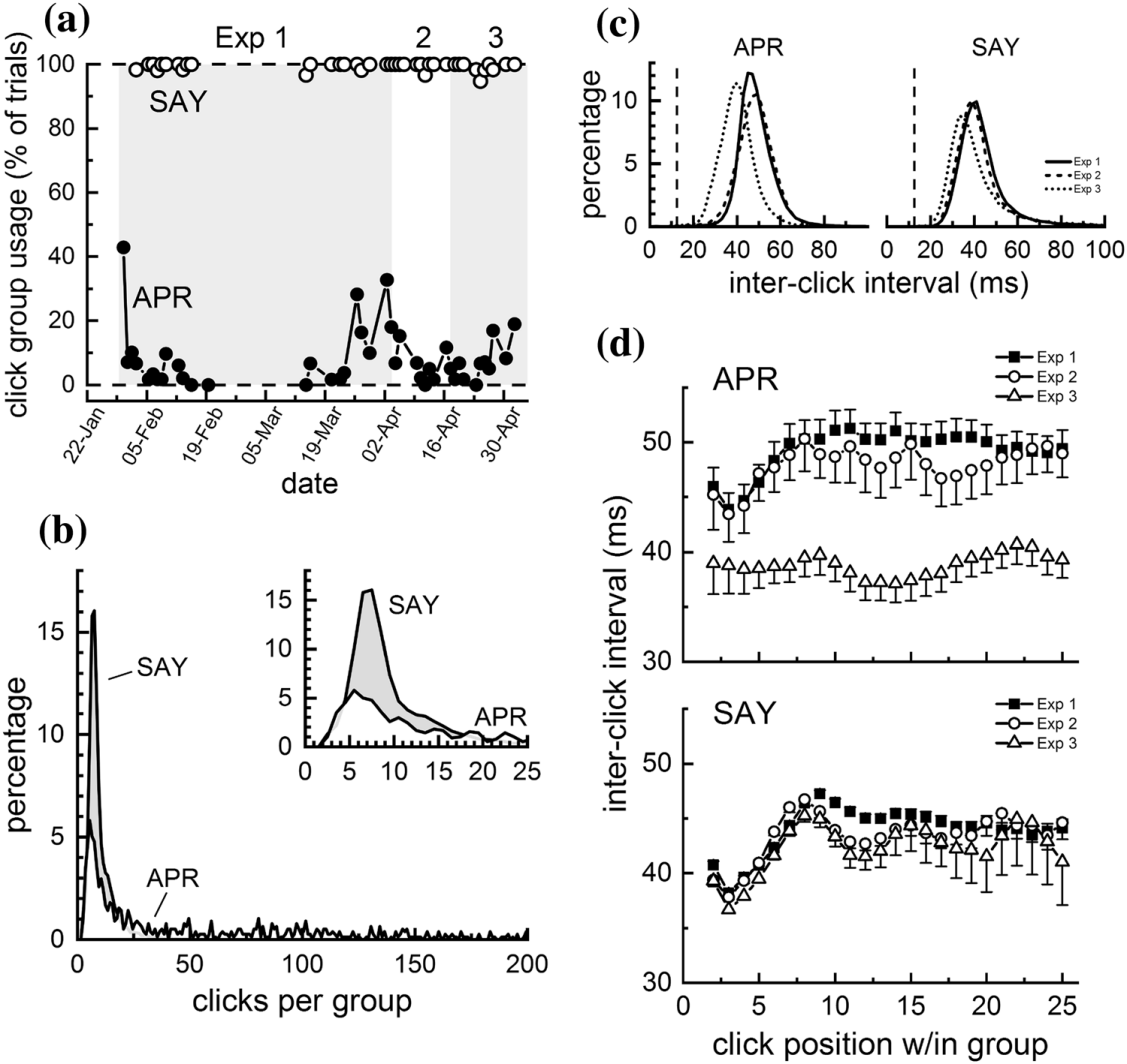
**a** Proportion of trials within each session that the dolphins APR and SAY utilized intermittent click groups (ICGs) during Exps. 1, 2, and 3. SAY almost always utilized ICGs, but APR used ICGs less often and more sporadically. **b** Distribution of the number of clicks within each intermittent click group (ICG) for the dolphins APR and SAY. The inset highlights the region from 0 to 25 clicks/group. For SAY, ICGs normally contained 5–10 clicks, but for APR, the number of clicks per ICG was more broadly distributed and some ICGs contained hundreds of clicks. **c** Inter-click intervals for the dolphins APR and SAY for Exps. 1, 2, and 3. The vertical dashed line shows the echo delay for the non-jittering echoes (i.e., the two-way travel time for the simulated echoes). Inter-click intervals always exceeded the echo delay. **d** Inter-click intervals as functions of click position within each intermittent click group (ICG), for the dolphins APR (upper panel) and SAY (lower panel). For both dolphins, there was often a tendency for the ICI to increase over the first 5–10 clicks within each ICG; i.e., the click rate tended to slow down within each ICG

Dolphins participating in short-range (i.e., < ~ 75 m) biosonar tasks similar to that of the present study typically produce continuous streams of clicks, with inter-click intervals (ICIs) exceeding the acoustic two-way time (TWT) from the dolphin to the target by a few tens of milliseconds (e.g., Finneran 2013; Penner 1988). This pattern is exemplified by the click train in Fig. 8a, where APR consistently produced clicks during the entire trial, with no temporal gaps and ICIs exceeding the TWT (i.e., the echo delay) by ~ 11–26 ms. In contrast to this typical pattern, both dolphins in the present study produced clicks using intermittent patterns, where relatively large temporal gaps separated groups of clicks (referred to here as “intermittent click groups”, ICGs, Fig. 8b–f), with ICIs always exceeding the TWT. Use of ICGs varied by dolphin, with SAY almost always utilizing ICGs and APR using ICGs only occasionally (Fig. 9). SAY began using ICGs about 3 months after training with jittered echoes began (about 4 months before Exp. 1 data collection); over the course of a few sessions, the ICG pattern changed from a few large ICGs within a trial (Fig. 8e, f), to more ICGs, each with fewer clicks, per trial (Fig. 8g–l). For SAY, ICGs normally contained 5–10 clicks, but for APR, the number of clicks per ICG was more broadly distributed and some ICGs contained hundreds of clicks (Fig. 9b). Within ICGs, there was often a tendency for the ICI to increase over the first 5–10 clicks; i.e., the click rate tended to slow down within each ICG (Fig. 9d), except for APR during Exp. 3.

## Discussion

### Jitter delay resolution

The goal of jittered echo delay acuity measurements is to measure the inherent delay sensitivity of the biosonar system, not to measure how accurately moving animals can use their biosonar to locate/track prey, etc. in natural environments. The jittered delay stimuli are therefore artificial in nature and designed to emphasize changes in echo delay while minimizing additional cues, not to replicate echoes from actual physical objects (see Simmons et al. 2003). In this respect, the paradigm is similar to the use of pure tones—idealized stimuli that bear little resemblance to communication or echolocation sounds—for determining auditory sensitivity, frequency discrimination limens, etc.

Results from Exps. 1 and 2 were similar and showed the 50% correct jittered echo delay thresholds in the dolphins APR and SAY to be 1–2 µs—significantly lower than the ~ 40 µs thresholds previously reported for a dolphin during range difference discrimination utilizing physical targets at 7 m (the closest to the 9.4-m simulated range in the present study, Murchison 1980). The present results are of the same order of magnitude as those reported for bats by investigators that did not test jitter delays below a few hundred nanoseconds (~ 0.4–0.5 µs, Menne et al. 1989; Moss and Schnitzler 1989; Simmons 1979). It is possible that experimental hardware with sub-microsecond resolution may reveal jitter detection thresholds in dolphins lower than those reported here (see “Methodological constraints”, below).

### Comparison with correlation functions and optimal receivers

A goal of the jitter experiments with bats was to determine if the bats’ jitter discrimination data resembled the XCR functions of the echoes (Simmons et al. 1990a). Using symmetric jitter delays, Simmons (1979) and Simmons et al. (1990a) found that the jitter discrimination curves approximated the half-wave rectified XCR function of the echoes, including a secondary peak in the error function near 30–35 µs.

The present data show close agreement between the dolphin click ACR and jitter delay error functions (Fig. 4), and the minimum resolvable jitter delays of 1–2 µs are well within the envelope of the click autocorrelation function. With symmetrical jitter (Exp. 1), both dolphins exhibited a secondary peak in the error function between 12 and 16 µs, qualitatively similar to error function peaks observed by Simmons (1979) and Simmons et al. (1990a), and roughly matching time lags at which secondary peaks occurred in the dolphin click ACR functions. With asymmetric jitter, both dolphins exhibited a drop in hit rate at 14 µs, but the corresponding peak in the error function was not well defined for the dolphin APR (due to higher error rates at adjacent jitter delays). Three previous studies using asymmetric jitter with bats also did not reveal secondary peaks in error functions (Menne et al. 1989; Moss and Schnitzler 1989; Simmons et al. 1990a). In two of the studies, overall discrimination performance tended to be lower when using asymmetric jitter and it was therefore possible that the overall higher error rates may have obscured the secondary peak in the error function, as seen in the present data for APR (Moss and Schnitzler 1989; Simmons et al. 1990a). It may also be possible that subtle differences in the experimental apparatus resulted in small performance drops at some jitter delays in some, but not all, studies (Menne et al. 1989). For both symmetric and asymmetric jitter conditions, jitter delay resolution measured for the dolphins (1–2 µs) was worse than that predicted for a coherent or semicoherent optimal receiver operating with similar estimated bandwidth and SNR (~ 2–80 ns for coherent and 30–300 ns for semicoherent, depending on the method used to estimate SNR).

When jittering both echo delay and echo polarity, Simmons et al. (1990a) reported that the peaks in the error function moved along the jitter delay axis according to the average period of the biosonar pulse. In the present data (Exp. 3), this was not seen for either the main error peak about 0 µs or the secondary peak between 10 and 15 µs, as there were no apparent peaks in the error functions. The error functions for both dolphins during Exp. 3 were more variable than those for the other two experiments, and it is possible that any peaks may have been obscured by the higher error rates. It is also possible that the larger separation of jitter delay values above 4 µs prevented a peak in the error function from being detected (i.e., better resolution in the jitter delay values may have revealed a peak).

Despite general agreement of biosonar data with models featuring a cross-correlation process, important questions have been raised as to whether correlation comparisons with farfield, on axis signals are appropriate (see Schnitzler et al. 1985). For dolphins (and other odontocetes) it seems particularly important to question the use of the farfield click as a template with which returning echoes are compared. Measurements of auditory evoked potentials during echolocation have shown that the dolphins’ emitted biosonar click (the “self-heard” click) is audible (e.g., Supin et al. 2003), and thus available as a template. However, the extent to which the self-heard click differs from the farfield click is not clear. Recent data show that neural responses to the self-heard click exhibit limited latency changes with frequency compared to the farfield click (Finneran et al. 2017), suggesting that there may be substantial differences between the farfield click and the dolphin’s internal representation of its outgoing click. In this respect, dolphins may be fundamentally different than bats, since the propagation path for the self-heard click in dolphins may primarily traverse cranial tissues (and air spaces) rather than the surrounding medium.

### Detection of polarity shift

Both dolphins were able to discriminate between echoes that changed only in polarity (also observed in *Eptesicus* by Menne et al. 1989; Simmons et al. 1990a), which superficially implies a sort of phase sensitivity. Although the high-frequency limit of phase locking in auditory neurons is not known for dolphins, it is likely that phase locking is roughly comparable to other mammals and not possible above several kilohertz (Heil and Peterson 2015). The results are nonetheless consistent with a model of half-wave rectification of the dolphin’s click and echo, followed by the delay difference detection capabilities (modeled using the XCR function) observed in Exps. 1 and 2. For a dominant click/echo period of 10–15 µs, an inversion of polarity results in a 5–8 µs shift in the timing of condensation/rarefaction peaks in the acoustic signal and thus in the peak firing of primary auditory neurons. This is well above the 1–2 µs limit of jitter delay resolution from Exps. 1 and 2 and would likely be detectable without requiring phase sensitivity per se.

The near 100% correct performance at 0 µs jitter with a polarity inversion contradicts the conclusions of Ibsen et al. (2013). In that study, a bottlenose dolphin was trained for a go/no-go procedure with a phantom echo generator. The “go” target was a simulated echo from a solid steel sphere, and the “no-go” targets were filtered versions of that echo. Full-spectrum target echoes with the polarity inverted (i.e., 180° phase shifted) were also tested. The dolphin responded to 40% of these targets, as compared to 100% for the standard targets and 0% for many of the filtered targets. The authors concluded that the dolphin was not sensitive to the phase of the echo. An alternative explanation, however, is that the intermediate response rate to the phase-shifted echoes (i.e., between the standard and filtered rates) indicates that the dolphin classified the polarity-shifted echo as unlike either of the other two echo classes. That the 40% response rate differed markedly from both the “go” and “no-go” targets seems to suggest that the dolphin may have detected a difference in the echo but did not reliably classify it as either. If this is indeed the case, then the present results may not contradict those from the earlier study. It is also possible that the echo-delay resolution required for detection of the polarity change was not available due to the experimental design of the Ibsen et al. (2013) study; i.e., it was not a jittered echo paradigm (Altes 1989).

### Click emission patterns

At times, both dolphins utilized ICGs, with SAY almost always utilizing ICGs and APR using ICGs only occasionally. The ICGs superficially resembled click “packets” utilized by dolphins or belugas performing long-range echolocation tasks (Finneran 2013; Ivanov 2004; Turl and Penner 1989); however, click packets utilized at long-range feature within-packet ICIs well under the TWT and between-packet intervals exceeding the TWT. In the present study, ICIs always exceeded the TWT. The click rate tended to slow down within each ICG, in contrast with the click-packet behavior exhibited by SAY during a previous long-range task, where click rate increased across each packet (Finneran 2013). The reasons for the unusual click emission patterns are not known. Dolphins are known to utilize multi-echo processing (Altes et al. 2003). It may be possible that combining information across jittering and non-jittering echoes created problems for the discrimination task, thus the dolphins improved performance by periodically interrupting click emissions and utilizing each ICG as an independent “look” at the target. How jittering the echo delay might affect the representation of the target within (and across) ICGs, e.g. “blurring” of the image, is unknown. It is also possible that the click emission patterns were related to some (unknown) feature of the acoustic environment.

### Methodological constraints

Although the general experimental approach was based on that of Simmons (1979), several modifications were made to better adapt the method to the relatively large subjects and the aquatic environment. Rather than a two-alternative forced-choice approach with spatially separated click receivers and echo projectors, a single click receiver and a single echo projector, both located on the dolphin’s main biosonar transmit axis, were used in conjunction with a go/no-go echo-change detection task. A contact hydrophone was used for the click receiver to reduce errors from relative motion between the dolphin and the click receiver. This approach had the advantage of not requiring the dolphin to move when inspecting the targets, and the single phantom echo system eliminated the potential for systematic errors due to differences in the left/right sound reception/transmission hardware and acoustic transmission paths. Comparison of the contact hydrophone and farfield clicks (Fig. 6) shows that the contact hydrophone affected the waveform of the received click by extending the time duration of the exponential decay (i.e., there were more cycles in the contact hydrophone signal compared to the farfield click). However, the spectral content and ACR functions were similar for the farfield and contact hydrophone clicks. The chief limitation of the present PEG system was that the echo-delay resolution was constrained by the D/A sampling interval (1 µs), therefore jitter delay acuity in the sub-microsecond range could not be tested.

Although echo delay relative to click reception could be precisely controlled (within the sampling interval limits), the time of click reception was taken as the time at which the sampled acoustic pressure crossed an amplitude threshold. The threshold was selected with the intent to trigger on the first full negative half-cycle of the received click (about 28 µs in Fig. 6, top row). In practice, the maximum potential changes in echo delay with fixed-amplitude, dolphin-like click signals were found to be small, less than ± 15 ns. However, since the clicks were digitally sampled, the actual threshold-crossing time could fluctuate up to 1/2 the sampling interval (± 0.5 µs), depending on the click waveform, potentially creating small, unintended changes in echo delay. If click amplitude changed substantially within a trial, it was possible for the threshold-crossing to vary, and even to occur on a different click cycle than intended. For example, if the click amplitude was too small for triggering to occur on the first negative half-cycle, triggering could occur on the second half-cycle, causing the echo delay to increase compared to the previous click. Such inadvertent changes in echo delay would have values near the dominant period in the click, between ~ 10 and 15 µs (based on the click autocorrelation functions). It is possible that this phenomenon contributed to the drop in hit rate seen in both subjects near jitter delays of 12–16 µs in Exps. 1 and 2, or to the dolphins’ use of ICGs. At the very least, the potential for unintended changes in echo delay would likely have made the task more difficult—the task was essentially discriminating controlled jittering echo delays presented among smaller occasional, random changes in echo delay. Similarly, relative motion between the dolphin and echo projector could have also caused apparent changes in echo delay and increased the task difficulty (although these movements were likely small, see “Methods”). Consideration of these potential errors (along with the 1-µs hardware sampling rate) suggests that the “true” jittered echo-delay resolutions may be smaller than those reported here. Analog delay lines, a digital filter with single tap along with a switching network, higher sampling rates, or a fractional delay filter would be necessary to reduce uncertainties in echo-delay resolution, although it should be noted that the potential for artifacts associated with analog delay lines has been highlighted (e.g., Beedholm and Mohl 1998).

## Conclusions

Bottlenose dolphins are able to detect jittered echo delay down to at least 1–2 µs, an order of magnitude less than the delay resolution capabilities measured in target range discriminations (Murchison 1980). Whether dolphins can resolve sub-microsecond jittered delays (as observed in bats) would require higher digital sampling rates or analog delay lines. Testing of jitter delays in the range of tens to hundreds of nanoseconds, at a variety of SNRs, would be required to determine if a coherent or semicoherent receiver model is appropriate for dolphins. Click patterns used by the dolphins differed from the typical continuous click trains observed in most short-range phantom echo tasks by including ICGs interspersed with pauses. The function of these click patterns is currently unknown, but they may facilitate increased jittered delay discrimination though a “resetting” of the memory of target characteristics.
